# Host Proteins Ku and HMGA1 As Participants of HIV-1 Transcription

**Published:** 2016

**Authors:** O. A. Shadrina, E. S. Knyazhanskaya, S.P. Korolev, M. B. Gottikh

**Affiliations:** Faculty of Bioengineering and Bioinformatics, Lomonosov Moscow State University, Leninskie Gory, Moscow, 119991, Russia; Chemistry Department, Lomonosov Moscow State University, Leninskie Gory, Moscow, 119991, Russia; Belozersky Institute of Physical-Chemical Biology, Lomonosov Moscow State University, Leninskie Gory, Moscow, Russia; 119991

**Keywords:** cellular transcription factors, latent phase, HIV-1 transcription, elongation

## Abstract

Human immunodeficiency virus type 1 is known to use the transcriptional machinery of the host cell for viral gene transcription, and the only viral protein that partakes in this process is Tat, the viral trans-activator of transcription. During acute infection, the binding of Tat to the hairpin at the beginning of the transcribed viral RNA recruits the PTEFb complex, which in turn hyperphosphorylates RNA-polymerase II and stimulates transcription elongation. Along with acute infection, HIV-1 can also lead to latent infection that is characterized by a low level of viral transcription. During the maintenance and reversal of latency, there are no detectable amounts of Tat protein in the cell and the mechanism of transcription activation in the absence of Tat protein remains unclear. The latency maintenance is also a problematic question. It seems evident that cellular proteins with a yet unknown nature or role regulate both transcriptional repression in the latent phase and its activation during transition into the lytic phase. The present review discusses the role of cellular proteins Ku and HMGA1 in the initiation of transcription elongation of the HIV-1 provirus. The review presents data regarding Ku-mediated HIV-1 transcription and its dependence on the promoter structure and the shape of viral DNA. We also describe the differential influence of the HMGA1 protein on the induced and basal transcription of HIV-1. Finally, we offer possible mechanisms for Ku and HMGA1 proteins in the proviral transcription regulation.

## INTRODUCTION

Although the human immunodeficiency virus (HIV- 1) was discovered over 30 years ago, the fight against the HIV infection still has not been won. Highly active antiretroviral therapy that is used to manage the HIV infection has significantly reduced mortality among patients with AIDS; however, interruption of treatment inevitably results in viral reproduction and increases the viral titer. One of the reasons for this phenomenon is the presence of cells in the human organism with the transcriptionally silent provirus integrated in their genome. The silent state, which is typical for the latent phase of the viral infection, is characterized by the absence of full-fledged transcription from the viral promoter. However, without treatment, the silent provirus can be activated and cause the development of AIDS [1].

The hairpin structure located at the 5’-end of the synthesized mRNA and known as TAR (trans-activation response) plays a key role in active transcription from the HIV-1 promoter. Elongation of transcription of the integrated viral genome takes place only when TAR is bound by the viral regulatory protein Tat (trans-activator of transcription) [2]. Formation of the TAR-Tat complex ensures phosphorylation of RNA polymerase II that is required for the elimination of transcription block and transition into the elongation stage [3]. However, Tat protein is not detected in the latently infected cells; hence, the mechanism of transcription activation of the silent integrated provirus upon transition from the latent into the lytic phase of the HIV-1 life cycle remains unclear. This is highly relevant to study the proteins partaking in the activation of transcription from the HIV-1 promoter via the Tatindependent mechanism, since in the long run it could allow one to understand the mechanism of transition of the virus from the latent to the lytic phase and to develop approaches to regulate this process.

It has recently been shown that cellular protein HMGA1 can be recruited in the regulation of transcription from HIV-1 promoter during the latent phase (basal transcription) [4, 5]. DNA- binding protein Ku, a component of DNA-dependent protein kinase (DNAPK), can also be involved in transcription regulation [6-10]. In this review, we summarize the data on the effect of the Ku and HMGA1 proteins on HIV-1 transcription and present the putative schemes for a possible involvement of these proteins in the regulation of transcription.

## REGULATION OF TRANSCRIPTION FROM THE HIV-1 PROMOTER

Human immunodeficiency virus type I is a member of the genus Lentivirus, part of the family Retroviridae. It affects the human immune system and causes the acquired immune deficiency syndrome (AIDS). Like the genome of other lentiviruses, the HIV-1 genome is an RNA molecule, which serves as a template for the synthesis of a DNA copy by viral enzyme reverse transcriptase. The DNA copy is then integrated into the cellular genome forming proviral DNA. However, most of the viral DNA remains non-integrated [11]. This DNA mainly exists in the circular form. Transcription can be carried out from the circular viral DNA, but it is the integrated provirus that serves as the main template for synthesizing viral proteins [12].

Being integrated into the chromosome of an infected cell, viral DNA can either stay silent or be actively transcribed. In other words, the transcription level can be low thus resulting in a small number of transcripts without rapid progression of the infection and is generally referred to as basal (not activated) transcription. Alternatively, transcription can be active and yield a large amount of RNA and new viral particles. Regulation of the HIV-1 genome transcription, which precludes the fate of the provirus, depends on a large number of factors: cis-acting elements of viral DNA, cellular transcription factors, viral trans-activator Tat, and the degree of chromatin condensation.

**Fig. 1:**
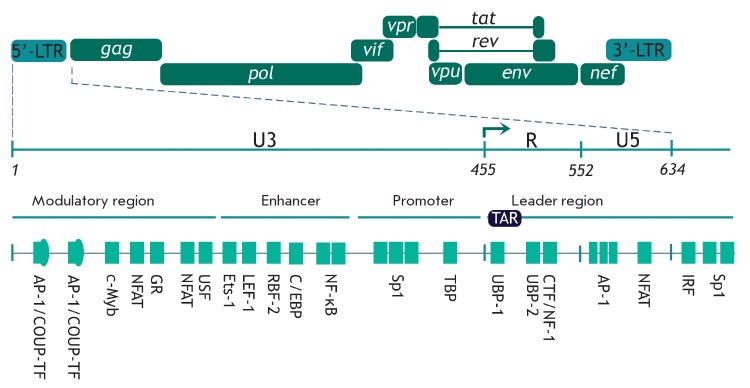
Binding sites of transcription factors in HIV-1 5’-LTR. Schematic representation of HIV-1 provirus and the major binding sites of transcription factors. Positions of the 5’-LTR regions are specified: U3 (nucleotides 1–455), R (456–552), and U5 (553–634). The transcription initiation site is shown with an arrow and corresponds to the border between the U3 and R regions [13].

Viral DNA integrated into the cellular genome carries long terminal repeats (LTRs) at its ends. Each of them consists of U3, R, and U5 regions (*Fig. 1*). Transcription starts at the border between the U3 and R regions in the 5’-LTR, since the viral promoter recognized by RNA polymerase II (RNAP II) and some other regulatory elements are located in the U3 region. 5’-LTR contains four functional regions partaking in the regulation of the HIV-1 genome transcription: the modulatory region, the enhancer, the promoter, and the leader regions (*Fig. 1*) [13]. They contain many binding sites for the cellular transcription factors, including the ones that play a crucial role in the transcriptional regulation: NF-κB, NFAT, Sp1, and AP-1 (*Fig. 1*). These factors are involved in the initiation of transcription [1, 13].

Transition of the provirus from a silent to an active state starts with the transcription initiation. Short abortive transcripts ~60–80 nucleotides long are synthesized [14]; they form a stable hairpin called TAR at the 5’-end. Just after the TAR RNA synthesis RNAP II stops, since it is associated with the factors that repress elongation: NELF (negative elongation factor) and DSIF (5,6-dichloro-1-β-*D*-ribofuranosylbenzimidazole sensitivity-inducing factor) [15]. To continue transcription and proceed to the active elongation stage, the C-terminal domain (CTD) of RNAP II needs to be hyperphosphorylated at Ser2 residues in heptapeptide repeats YSPTSPS. Hyperphosphorylation is ensured by the transcription elongation factor P-TEFb (positive transcription elongation factor b), which consists of cyclin- dependent kinase 9 (Cdk9) and cyclin T1 (CycT1). The level of accessible P-TEFb is regulated by its binding to 7SK small nuclear ribonucleoprotein (7SK sn- RNP), which inhibits the kinase activity of the P-TEFb factor and impedes transcription elongation [16].

**Fig. 2:**
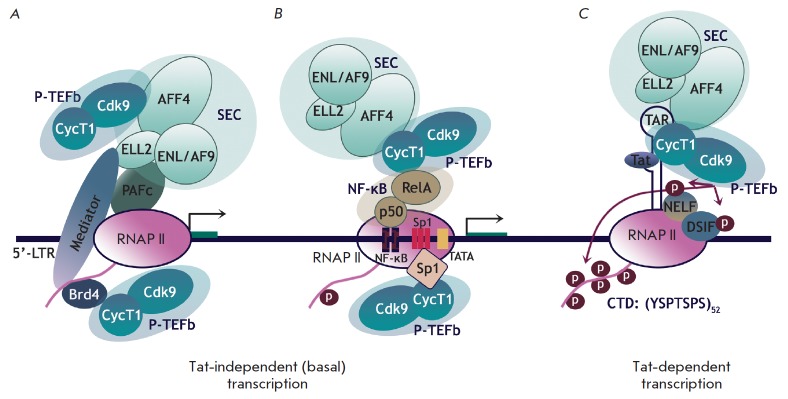
Possible mechanisms for recruitment of P-TEFb to the HIV-1 promoter. *A, B *– Tat-independent transcription when P-TEFb stimulates basal transcription from the HIV-1 promoter in the absence of Tat. P-TEFb can be recruited by cellular proteins Brd4, SEC (*A*) or NF-κB, Sp1 (*B*). *C – *Tat-dependent transcription. Tat is bound to TAR RNA, thus facilitating the release of P-TEFb from 7SK nsRNP and its recruitment to the paused elongation complex [14].

The viral protein Tat is the key regulator at the elongation stage: it enhances efficiency of RNA synthesis by several orders of magnitude [2]. Binding of Tat to the synthesized TAR RNA facilitates dissociation of PTEFb from the complex with 7SK snRNP and recruits it to the viral promoter. As a result, P-TEFb ensures hyperphosphorylation of RNAP II, as well as the NELF and DSIF factors [17]. Phosphorylation of DSIF converts it to the activating elongation factor, while phosphorylated NELF dissociates from the transcription complex, thus allowing RNAP II to perform effective elongation and synthesize full-size mRNA (*Fig. 2*) [3].

However, Tat is not detected in cells at the latent stage of infection. Neither is it found when the provirus starts exiting from dormancy. In some cases, the TAR– Tat–P-TEFb complex cannot be formed due to mutations disrupting the interplay between its components [18]. Nevertheless, transcription from the HIV-1 promoter may still occur. Several mechanisms of Tat-independent activation of transcription are known. First, it has been assumed that P-TEFb can perform phosphorylation of the CTD RNAP II required for transcription elongation in the absence of Tat [19]. Some cellular factors (Sp1 [20], SEC [14], Brd4 [21, 22], and NF-κB [14]) probably participate in the recruitment of P-TEFb to the viral promoter (*Fig. 2A,B*). Alternatively, a cellular protein different from P-TEFb but capable of the phosphorylation of CTD RNAP II (as well as the NELF and DSIF repressive factors) may bind to the HIV-1 promoter.

Although a significant amount of data on the regulation of HIV-1 transcription and involvement of various cellular factors in it has been accumulated, many aspects have not been completely elucidated yet. In particular, the role of two cellular proteins involved in HIV-1 transcription (Ku and HMGA1) remains unclear. Some available data attest to the positive role of these proteins in the regulation of transcription, while other studies demonstrate that their role is negative. Nevertheless, the accurate mechanisms of involvement of these proteins in HIV-1 transcription are still to be determined.

## ROLE OF KU PROTEIN IN HIV-1 TRANSCRIPTION

Human Ku protein is a heterodimer consisting of two subunits with masses of ~70 and 80 kDa, which are known as Ku70 (p70) and Ku80 (Ku86, p80). These proteins are encoded by the *xrcc6 *(Ku70) and *xrcc5 *(Ku80) genes. Ku protein mainly functions in the cell in the form of a very stable heterodimer [23]. However, some research demonstrates that isolated Ku70 and Ku80 subunits can be involved in certain processes [24].

**Fig. 3:**
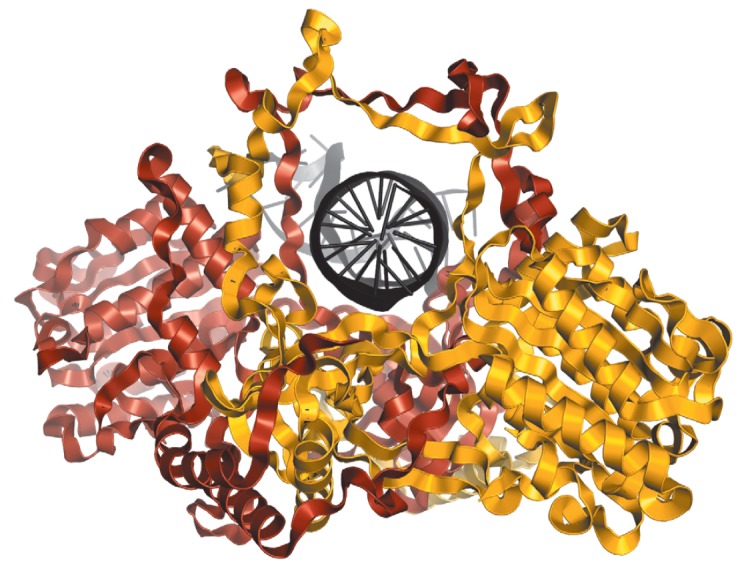
Structure of the Ku heterodimer in a complex with DNA according to [26]. DNA (shown in black) resides in the channel formed by Ku70 (shown in yellow) and Ku80 (shown in brown). PDBID 1JEY

The Ku70/Ku80 heterodimer is a DNA-binding protein that mostly interacts with the free ends of doublestranded DNA, and its biological function is mainly related to this feature. The interaction between the Ku heterodimer and DNA is rather strong: the *K*d value varies within a range of 1.5–4.0 × 10^-10^ M [25]. According to X-ray data [26], Ku70 and Ku80 within a heterodimer form an asymmetric ring with a wide base and a thin bridge; the resulting channel is big enough to encircle DNA (*Fig. 3*). The channel predominantly consists of positively charged amino acid residues that interact with the negatively charged sugar-phosphate backbone of the DNA molecule, which explains why Ku can bind DNA in a sequence-independent manner. After binding to the DNA end, Ku can migrate (slide) along DNA and pause at certain sequences [25, 27].

The most well-known and the best studied biological function of Ku is its involvement in double-strand DNA break repair by nonhomologous end joining (NHEJ). Ku also participates in such cellular processes as V(D) J-recombination, mobile element-induced genomic rearrangement, telomere length maintenance, apoptosis, and transcription [28, 29]. One key function of Ku is binding to the DNA-PKcs catalytic subunit to form DNA-dependent protein kinase DNA-PK. It is worth mentioning that the catalytic function of DNA-PK is activated after its binding to DNA, which is provided by the Ku heterodimer [25].

**The possible mechanisms of Kumediated regulation of transcription**

Participation in transcriptional regulation is one of the numerous functions of Ku. Several mechanisms of transcriptional activation or suppression by Ku have been described. The first mechanism is a direct sequence- specific interaction between Ku and the promoter region of genes. It has been hypothesized that transcription of the cellular genes *c-Myc, Hsp70* [30], U1 snRNA [31], as well as retroviruses HTLV-1 (human T-lymphotropic virus) [32] and MMTV (mouse mammary tumor virus) [33], is regulated via this mechanism. The mechanism of Ku binding to the promoter region that does not involve interactions with DNA ends is yet unclear; however, there is data attesting to a possible sequence-specific interaction between the heterodimer and a certain Ku-binding motif in DNA [34].

**Fig. 4:**
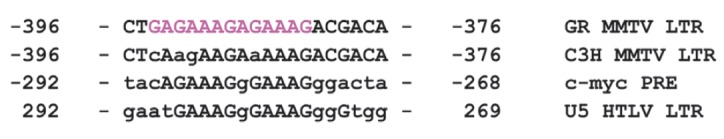
Putative Ku-binding sites in gene promoters. Ku-binding sites homologous to the NRE-1 sequence in the LTR of the GR strain of MMTV. Direct repeat is shown in color. Mismatches are denoted by lowercase letters [34].

A sequence whose binding to Ku is considered to be truly sequence-specific and more preferable compared to Ku binding to DNA ends has been identified in the NRE-1 region (negative regulatory element 1) in the LTR of MMTV retrovirus (*Fig. 4*) [34]. The interaction between Ku and this sequence reduces the efficiency of transcription from viral LTR. The catalytic subunit DNA-PKcs is believed to be involved in this regulation [33, 35]. It has been demonstrated that GR (glucocorticoid receptor) [34] and Oct-1 [36], the two transcription factors binding to 5’-LTR MMTV and activating its transcription, can be *in vitro *phosphorylated by DNAPK. Specific recruitment of DNA-PK to the promoter and subsequent phosphorylation of transcription factors is probably one of the transcriptional regulatory mechanisms.

All the Ku-binding sites in promoters homologous to the NRE-1 sequence in the promoter of the GR strain of MMTV and known up to the publication date are reported in [34] (*Fig. 4*). Only these sequences were shown to be capable of direct and specific interaction with the Ku heterodimer in the absence of free DNA ends.

The *second mechanism *via which Ku affects the transcription is its direct interaction with transcription factors, including Oct-1, Oct-2 [36], NF45/NF90 [37], AP-1 [38], Ese-1 [39], YY1 [40], and p53 [41]. Some of these factors are involved in the regulation of HIV-1 transcription. In addition, as mentioned above, some transcription factors can act as a DNA-PK substrate* in vitro*. The ability of DNA-PK to interact both with transcription factors and a number of nuclear receptors (AR [42], GR [34], PR [43], and ER-α [44]) probably suggests that there is a shared mechanism via which Ku participates in cell signaling and transcription regulation.

Ku can indirectly regulate gene transcription by influencing the expression of other transcription factors. Thus, in the AGS cell line, Ku positively regulates the expression of the gene of the NF-κB p50 subunit [45]. Ku80 also stimulates the expression of the *c-jun *gene, the AP-1 transcription factor component [38]. It should be mentioned that NF-κB and AP-1 are the key regulators of the transcription of HIV-1 genes.

The Ku70 and Ku80 subunits may have a different effect on transcription. It has been demonstrated that the subunits of the Ku heterodimer dynamically bind to the promoter of the interleukin 2 (IL-2) gene and interact with the NF45/NF90 factor in response to T-cell activation [37]. This activation increases the amount of the Ku80/NF90 complex bound to the antigen receptor response element (ARRE) sequence in the *IL-2 *gene promoter, while the amount of the Ku70 subunit bound to this region decreases [37]. In another work [30], the repressive role of Ku in the transcription of the *Hsp70* gene was attributed to the Ku70 subunit rather than to Ku80.

The *third mechanism *of transcriptional regulation is the direct interaction between Ku heterodimer or its Ku80 subunit and RNAP II holoenzyme. Ku80 was found to be colocalized with the elongational form of RNAP II and transcription factors specific for the elongation stage (in particular, DSIF) in the nucleus. The C-terminal domain of Ku80 was also found to play a key role in the interplay with these proteins [48]. Let us mention that DNA-PK can phosphorylate RNAP II *in vitro *[49]; however, the role of this phosphorylation in transcriptional regulation still needs to be ascertained.

The *fourth mechanism *of Ku involvement in transcriptional regulation is related to its role in the repair of double-strand DNA breaks [50]. Double-strand breaks need to be introduced by DNA topoisomerase IIβ to successfully initiate transcription from a number of promoters regulated by binding to AP-1 and nuclear receptors (including those interacting with Ku). In this case, the break repair and local alterations in the chromatin structure occur in the presence of the complex of the proteins PARP-1 (poly[adenosinediphosphate (ADP)–ribose]polymerase-1), DNA-PKcs, and Ku70/ Ku80.

Hence, the isolated heterodimer subunits, the heterodimer as a whole, or its complex with the DNAPKcs catalytic subunit can be involved in the regulation of transcription. No common mechanism of action of Ku has been revealed. It is most likely that there is a specific mechanism of Ku-dependent regulation for each particular gene. It should be mentioned once again that Ku may act both as an activator and as a suppressor; its effect usually depends on subunits, which are involved in the regulation. It seems that the catalytic subunit of DNA-PK is not necessarily involved in the Ku-dependent regulation of transcription; however, in certain cases its capability of DNA-dependent phosphorylation of transcription factors and RNAP II can be the key element of regulation.

**Role of Ku in HIV-1 transcription**

The significance of Ku for maintaining the HIV-1 life cycle has been demonstrated in numerous studies. The Ku70 subunit is a part of the pre-integration complex and interacts with HIV-1 integrase [51, 52]. The Ku80 subunit was detected within the virion [53], where it can be incorporated at the stage when a new viral particle is formed in a previously infected cell. Repair of single- strand breaks formed when viral DNA is integrated into the cellular genome is required for successful integration of viral cDNA into the host cell genome. It is believed that proteins from the NHEJ system, and the Ku70/Ku80 heterodimer in particular, can be involved in this process [54]. Thus, lentiviral vector transduction efficiency is significantly decreased in cells defective in Ku80, DNA-PKcs, Xrcc4 (X-ray repair cross-complementing protein 4), and DNA ligase IV [55, 56]. Ku is also involved in the formation of the circular form of viral DNA from non-integrated linear DNA [57-59].

Involvement of Ku in the transcriptional regulation of HIV-1 was first reported in the early 2000s. However, the role of Ku in this regulation is still to be clarified. Data attesting both to the positive and negative effects of Ku on transcription of the HIV-1 genome have been obtained.

The role of Ku in transcription from viral 5’-LTR was first studied using the xrs-6 cell line, a variant of CHO-K1 (Chinese hamster ovary) cells lacking *Ku80* gene expression. These cells supported enhanced expression from the plasmid carrying the *CAT *(chloroamphenicol acetyltransferase) gene under the control of the viral promoter from 5’-LTR [6]. Stable transfection of xrs-6 cells with the vector carrying the human *Ku80* gene reduced CAT expression. Hence, Ku80 has a negative effect on transcription from the HIV-1 5’-LTR promoter. The negative role of Ku80 has been confirmed using a human U1 cell line whose genome contained the integrated provirus; this cell line is used as a model of the latent state of HIV-1. It turned out that a decreased amount of endogenous Ku80 in the cells increases the level of transcription of the HIV-1 genes, both for basal and TNFα-induced transcription.

**Fig. 5:**
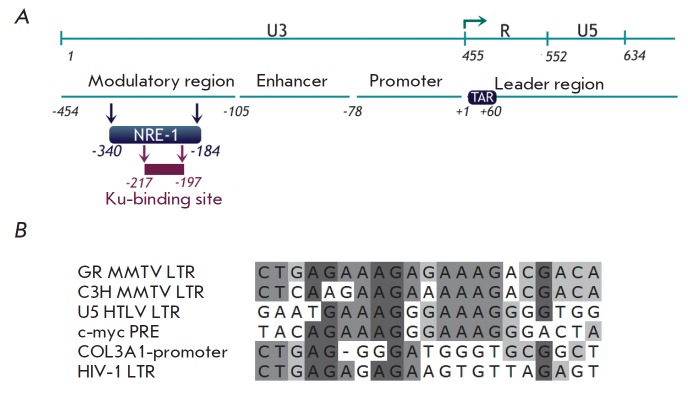
Scheme of the HIV-1 genome and position of the Ku-binding site in 5’-LTR. *A *– positions of the 5’-LTR regions are specified: U3 (nucleotides 1–455), R (456–552), and U5 (553–638) (counting from the beginning of the genome). The major regulatory regions of 5’-LTR are shown. +1 – counting from the transcription initiation site denoted by an arrow. The Ku-binding site predicted in [6] is shown. *B *– alignment between the putative Ku-binding sites in the NRE-1 region of HIV-1 and MMTV, as well as some other sequences similar to MMTV NRE-1 [6].

Taking into account that Ku has a negative effect on transcription from other retroviral promoters (MMTV, HTLV-1) [32, 33], L. Jeanson and J.F. Mouscadet [6] searched for the Ku-binding site in the HIV-1 LTR and detected a motif (-217/-197) in the NRE-1 region that was rather similar to the Ku-binding site in MMTV NRE-1 (*Fig. 5*) [6]. Several variants of Ku-mediated repression of HIV-1 transcription were proposed. Considering the fact that Ku can bind to the Oct-1 and Oct-2 transcription factors [36], which repress both the basal and Tat-activated transcription of HIV-1, it was hypothesized that binding of Ku in the modulatory region (*Fig. 5A*) facilitates recruitment of these factors to the HIV-1 promoter [6]. It is also possible that Ku can be involved in the regulation of the chromatin structure. NRE-1 contains a nuclear matrix binding site [60] overlapping with the predicted Ku-binding site. The interplay between Ku and the nuclear matrix in this region presumably impedes NF-κB-activated transcription.

In their next study [7], this group of authors reported that Ku80 negatively influences transcription from retroviral vectors. It turned out that regardless of the promoter used in the lentiviral system, transcription was more active in the absence of Ku80. In other words, the effect of Ku80 on retroviral vector expression was found to be not sequence-specific; hence, the Ku-binding site suggested in [6] could not completely explain the mechanism of negative transcriptional regulation. It has also been reported that Ku80 has no effect on the efficiency of transduction and integration of lentiviral vectors. Meanwhile, no Ku-dependent regulation was observed when plasmid vectors carrying the same promoters were used as a template instead of the pseudotyped virus [7]. Ku80 is believed to guide integration of lentiviral vectors into transcriptionally inactive regions instead of directly influencing transcription.

As opposed to the aforementioned findings, Ku plays a positive role in the regulation of transcription from the HIV-1 promoter in human MAGI and CEM-T4 cell lines [8]. Furthermore, insertion of siRNAs targeting Ku80 reduced the efficiency of integration of the viral genome into the infected cell DNA and disrupted Tat- TAR trans-activation.

The positive influence of the Ku heterodimer on transcription of the HIV-1 genome was also mentioned in [9], where wild-type HCT 116 human cells and their* Ku80*^+/-^ variant, which were transduced with HIV-1- based lentiviral vectors, were used. It has been preliminarily shown that in the HCT 116 *Ku80*^+/-^ cells with a twofold reduced level of Ku80 the level of Ku70 is equally lowered. It turned out that a twofold decrease in the amount of endogenous Ku in cells reduces the efficiency of viral transcription. It should be mentioned that, as opposed to data [7], this effect was specific for viral LTR, since changes in the Ku heterodimer level had no effect on transcription from other promoters. Moreover, viral proteins were not involved in Ku-mediated transcriptional regulation and the influence of Ku was independent of Tat-trans-activation. The effect of Ku on transcription was also noticeable in the presence of Tat; however, its effect was most significant at the basal level of transcription from the nonactivated provirus, when Tat is not detected in the cell. Interestingly, Ku influences the basal HIV-1 transcription only at the initial stage after integration of the viral genome and reduction of the Ku level in cells contributes to the emergence and maintenance of viral latency.

It is known that Ku80 is incorporated into the virion during its assembly [53]. Hence, both endogenous Ku80 and Ku80 from the virion can influence provirus transcription in the infected cell. In order to eliminate the effect of the latter, the lentivirus was harvested in the cell line with a decreased level of Ku heterodimer [9]. It turned out that it is endogenous Ku that affects transcription in the target cell.

It should be mentioned that the involvement of the previously predicted Ku-binding site in HIV-1 LTR in Ku-mediated regulation of the provirus transcription was also refuted in [9]. Replacement of this site with a random sequence had no effect on Ku-dependent transcriptional regulation.

Another important aspect of this study is that Ku does not affect transcription from the circular forms of viral DNA. Taking into account that, according to [7], Ku had no effect on transcription from the HIV-1 promoter within a plasmid vector, it can be concluded that Ku stimulates transcription only from the provirus integrated into the genome.

We would like to draw special attention to study [10] by S. Tyagi *et al*. who investigated the possible involvement of both the Ku70/Ku80 and the entire DNA-dependent protein kinase DNA-PK in the transcriptional regulation of HIV-1. Experiments were carried out using Jurkat-E4 cells whose DNA carried the integrated HIV-1 genome. Thus, this cell line was used as a model of T cells in the latency period of infection. DNA-PK was found on the HIV-1 promoter, and its location correlates with that of RNAP II. It was also determined that transcription activation significantly increases the DNA-PK and RNAP II levels not only on the promoter, but also on the transcribed region of the genome.

It has also been demonstrated [10] that DNA-PK can phosphorylate the C-terminal domain of RNAP II. Furthermore,* in vitro *experiments showed that DNA-PK predominantly phosphorylates Ser2 rather than Ser5 or Ser7 in the heptapeptide repeats YSPTSPS. Considering the fact that phosphorylation of Ser2 is required to activate elongation, it has been hypothesized that involvement of DNA-PK can be important, mostly at the elongation phase of transcription. DNA-PK presumably directly interacts with RNAP II at the HIV-1 promoter, and DNA-PK can act as a factor phosphorylating polymerase and eliminating elongation block. At any rate, parallel distribution of DNA-PK and RNAP II along the provirus and their simultaneous recruitment in response to transcription activation allow one to suggest that DNA-PK (and Ku as its component) is an element of a large transcriptional complex that is involved in HIV-1 gene expression.

Furthermore, it has been demonstrated that DNAPK has a positive effect on transcription from HIV-1 5’-LTR and lentiviral vectors [10]. Knockdown of the catalytic subunit DNA-PKcs in Jurkat cells significantly reduces expression of LTR-regulated genes and has a minor effect on expression from another promoter (CMV). Hence, knockdown of both DNA-PKcs [10] and Ku80 [9] reduces the level of transcription from the LTR promoter.

Summarizing the role of the Ku protein in the regulation of HIV-1 transcription, it should be mentioned that the currently available data are rather controversial. The possible reason is that different cell lines and different viral systems were used. Thus, most data attesting to negative regulation have been obtained using rodent cells, which obviously cannot be an adequate model for processes occurring in HIV-1-infected human cells. Nevertheless, the data obtained using human cells provide ground for drawing some reliable conclusions.

The first general conclusion is that Ku-mediated regulation of transcription depends on viral LTR. The regulation mechanism remains unclear, but one should not rule out the possibility of direct binding of the heterodimer to LTR, although the putative Ku-binding site within LTR probably is not the key element in Kudependent regulation of HIV-1 transcription, as opposed to MMTV and HTLV-1.

Second, the integrated provirus is crucial for Kumediated transcriptional regulation of HIV-1 and HIV-1-based lentiviral vectors. Although HIV-1 transcription can occur from circular DNA, it is clear that Ku is not involved in its regulation. This can be explained by the fact that the heterodimer is recruited to the provirus either during integration or immediately after it.

Let us mention once again that there remain many questions concerning the mechanism of involvement of Ku in the transcriptional regulation of HIV-1. Even if the incorporation of the Ku70/Ku80 heterodimer or the entire DNA-PK into the transcription complex is considered to be an established fact, the mechanism through which they influence HIV-1 gene expression has not been elucidated yet and it is important to clarify their role in HIV-1 transcription.

**Role of HMGA1 in HIV-1 transcription**

HMGA1 (high-mobility group protein A1, previously known as HMG I[Y]), the DNA-binding non-histone chromatin protein, is another cellular protein whose role in the HIV-1 life cycle has not been studied sufficiently. HMGA1 carries three DNA-binding motifs that preferentially bind to the DNA minor groove in ATrich regions (A/T hook) [61]. However, HMGA1 is more likely to recognize the spatial structure of DNA than the nucleotide sequence: it prefers to interact with bent and supercoiled DNA, with DNA that has a structure different from the classical B-form. The free protein has an unordered spatial structure. When interacting with DNA, it undergoes conformational changes, thus facilitating ATP-independent DNA unwinding, supercoiling, and bending [62, 63]. This ability to change the chromatin structure determines the broad range of functions performed by HMGA1 in the cell nucleus.

**Fig. 6:**
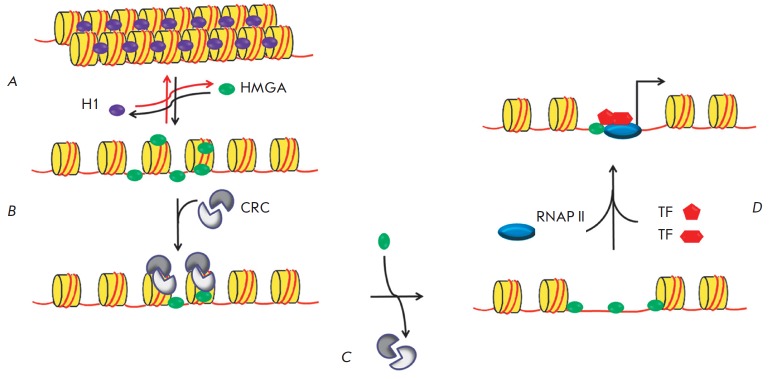
The putative model of HMGA1-mediated activation of transcription. The putative mechanism of transcriptional regulation by HMGA1: HMGA1 promotes chromatin reorganization by exposing DNA sites for transcription initiation factors. *A *– HMGA1 competes with histone H1 by replacing it. *B *– chromatin decompactization using chromatin-remodeling complexes (CRCs). Binding of CRC to chromatin increases when it interacts with HMGA1. *C *– release of DNA for binding to transcription factors. *D *– initiation of transcription: HMGA1 can interact with transcription factors (TFs) by recruiting them to the promoter [62].

Actually, all the high-mobility group proteins are capable of binding both DNA and proteins, which allows them to get involved in a large number of processes [64]. Alteration in the chromatin structure induced by HMGA binding either stimulates or represses such DNA-dependent processes as transcription, replication, and DNA-repair. HMGA1 is considered to be an architectural transcription factor, and this emphasizes its role in the organization of multiprotein complexes bound to the promoter [62-64]. The ability of HMGA1 to interact with core histones and displace the linker histone H1 from DNA results in chromatin reorganization and exposure of transcription factor binding sites (*Fig. 6*). HMGA1 plays a crucial role in the regulation of enhanceosome assembly or disassembly, thus affecting transcription. It has been repeatedly demonstrated that HMGA1 directly interacts with other chromatin-re-modeling proteins and transcription factors (Sp1, TFIID, NF-κB, ATF-2, SRF, Oct2, and c-Rel) [62, 63]. The ability of HMGA1 to bend DNA upon binding probably facilitates spatial proximity of the enhancer and promoter regions of the genes.

Involvement of HMGA1 in the life cycle of HIV-1 has been demonstrated in many studies. This protein was detected within the pre-integration complex [65]. HMGA1 was found to stimulate the integration of HIV-1 DNA into the cellular genome [66, 67]. It is assumed that HMGA1 binds to and bends viral DNA, pulling the ends together and facilitating their binding to integrase. Meanwhile, no direct interaction between HMGA1 and HIV-1 integrase has been observed. However, other authors have been critical of the idea that HMGA1 is involved in retroviral integration, since the absence of HMGA1 in infected cells had no effect on the integration of the viral genome [68].

Some rather ambiguous evidence in support of the involvement of HMGA1 in HIV-1 transcription has been obtained to date.

**Fig. 7:**
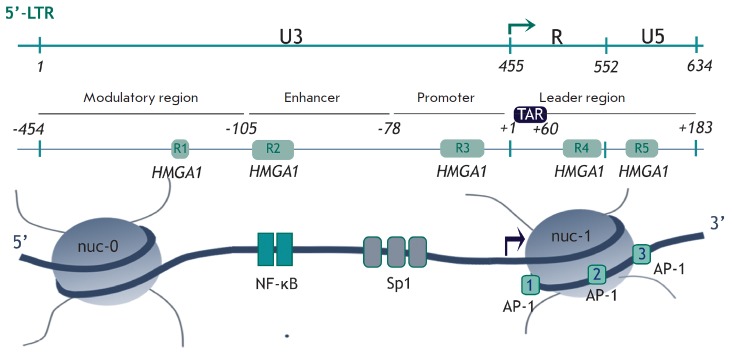
Positions of the predicted HMGA1 binding sites at HIV-1 5’-LTR. Positions of the 5’-LTR regions are specified: U3 (nucleotides 1–455), R (456–552), and U5 (553–634). Nucleotides are counted from the beginning of the genome. The major regulatory regions of 5’-LTR are shown. +1 – counting from the transcription initiation site denoted by an arrow. HMGA1 binding sites determined in [69] are shown. The positions of nucleosomes on the HIV-1 promoter are shown below; three sites of binding to the AP-1 transcription factor are specified.

Putative HMGA1 binding sites have been detected by DNase I footprinting in the -187/+230 region within HIV-1 LTR (R1–R5 in *Fig. 7*) [69]. The interplay between HMGA1 and the transcription factor AP-1 has also been studied: they were found to share a binding site (R5 in *Fig. 7*). This site resides at the border of the repressive nucleosome nuc-1, which exists in the provirus near the transcription initiation site in the latent phase and degrades after transcription of the viral genome is activated (*Fig. 7*). HMGA1 was found to facilitate binding of AP-1, an important inducible HIV- 1 transcription activator, to viral DNA in response to external stimuli activating viral expression. HMGA1 is possibly involved in the reorganization of nuc-1 by competing for this site and making it free for AP-1. Hence, it has been suggested that HMGA1 can positively regulate HIV-1 transcription [69].

The role of HMGA1 as an architectural transcription factor involved in the reorganization of nucleosome nuc-1 has been confirmed [70]. HMGA1 was found to facilitate binding of the ATF-3 subunit of the transcription factor AP-1 to site R3 at the border of nuc-1 in response to induction of viral transcription by PMA (phorbol myristate acetate – NF-κB activator) (*Fig. 7*). This makes it possible to recruit the ATP-dependent chromatin-remodeling complex SWI/SNF to the repressive nucleosome: a process required for efficient activation of viral transcription.

**Fig. 8:**
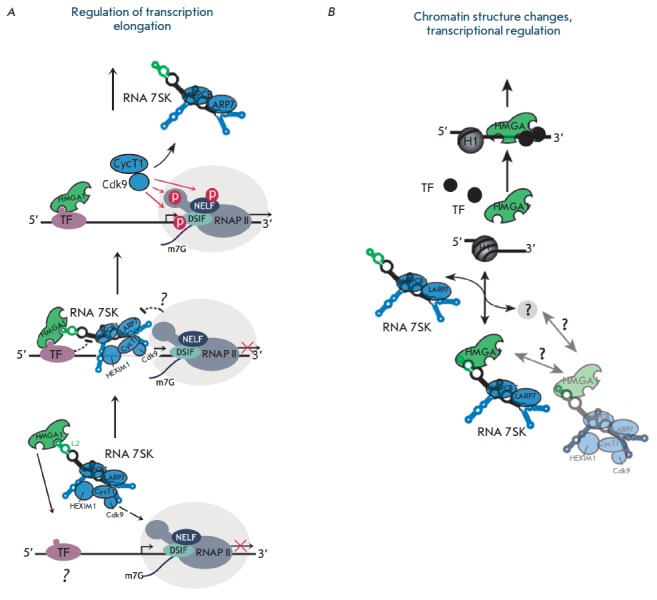
Mechanism of transcriptional regulation by the HMGA1–7SK–P-TEFb complex. *A *– HMGA1 can recruit P-TEFb/ 7SK snRNP complex to the paused transcription complex by interacting with DNA or a certain transcription factor. B – the level of free HMGA1 in the nucleus is regulated by its binding to 7SK snRNP. Dissociation of HMGA1 from its complex with 7SK snRNP can be caused by a factor that has not been identified yet [4, 72].

Another possible mechanism via which HMGA1 can be involved in transcription regulation has recently been proposed [71]. It turns out that HMGA1 binds to loop 2 of RNA in 7SK snNRP (7SK L2 RNA). As mentioned above, the key function of 7SK RNA is to regulate the level of the free P-TEFb factor activating transcription elongation [17]. This factor interacts with loop 1 and the HEXIM1 protein within 7SK snRNP. As a result, the HMGA1 complex with 7SK snRNP and P-TEFb can be formed. The role of this complex in transcription regulation can be a dual one (*Fig. 8*) [72]. First, HMGA1 can bind directly to DNA or a transcription factor located on the promoter region and recruit P-TEFb to the paused RNAP II elongation complex (*Fig. 8A*). Secondly, binding of 7SK to HMGA1 regulates the amount of free HMGA1 that can interact with DNA and functions in various processes (*Fig. 8B*). The mechanism is believed to depend on the nature of the particular gene.

In the case of HIV-1 transcription regulation, Sp1 can be a factor that interacts with HMGA1 and, thus, is recruited to the elongation complex. On one hand, this factor is known to be involved in HIV-1 transcription [1, 13], while on the other hand it directly interacts with HMGA1 [62]. Hence, upon transcription from HIV-1 LTR, HMGA1 may be involved in P-TEFb-dependent activation of elongation via the scheme shown in *Fig. 8A* [72] and, therefore, have a stimulating effect.

**Fig. 9:**
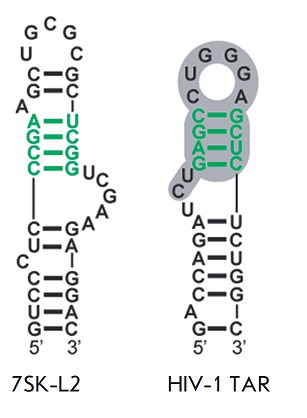
Structures of the 7SK L2 and TAR RNA regions interacting with HMGA1. The HMGA1 binding site in both RNAs is shown in green. The TAR region responsible for the interaction with Tat and CycT1 is shown in gray [4].

**Fig. 10:**
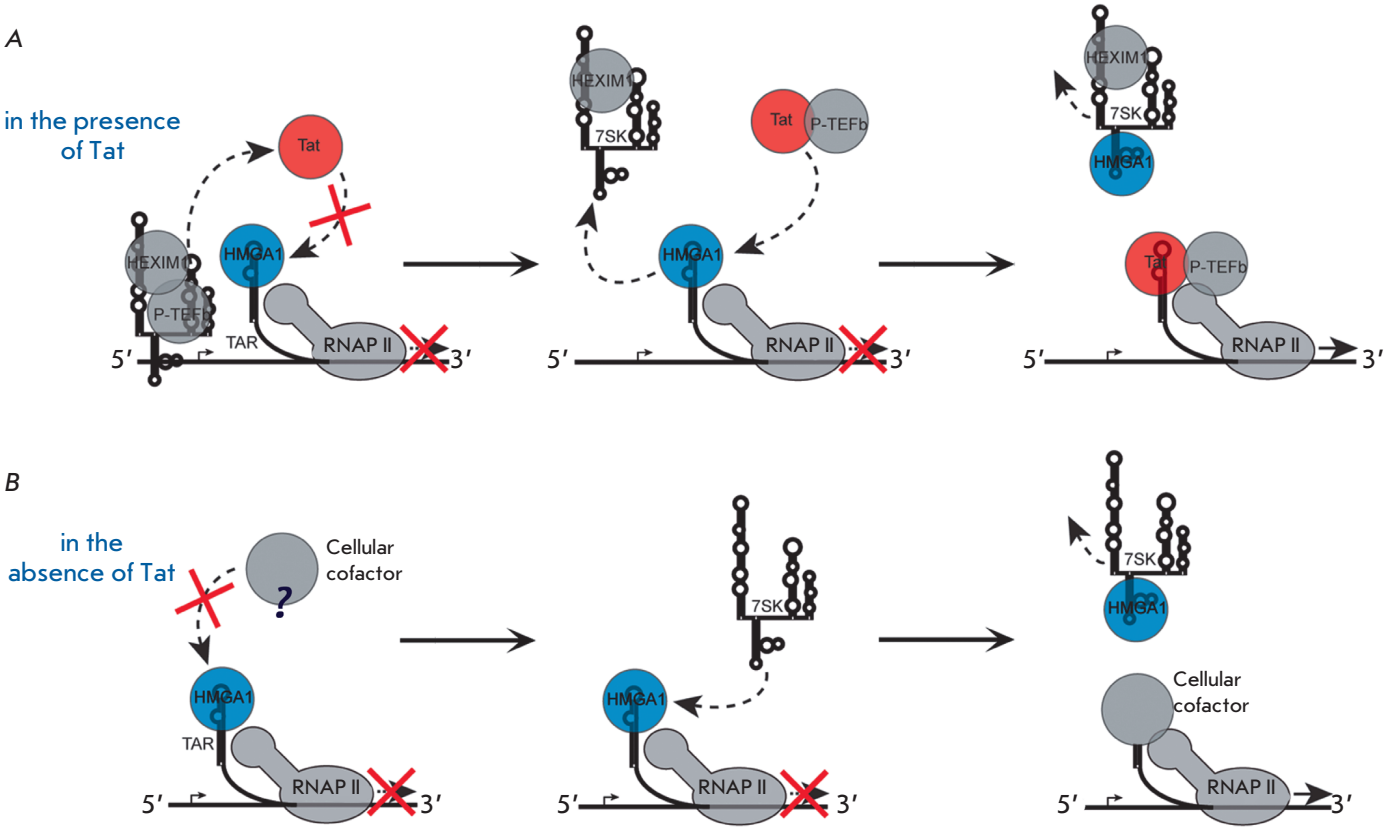
Model of HMGA-1-mediated repression of HIV-1 transcription. *A *– competition between HMGA1 and Tat for TAR reduces the activity of the viral promoter. Tat releases 7SK from its complex with P-TEFb bound to the promoter. 7SK binds to HMGA1 to release TAR for subsequent interaction with Tat-P-TEFb. *B *– in the absence of Tat, HMGA1 impedes binding of a certain cellular cofactor, which is required for TAR-mediated HIV-1 transcription, to TAR RNA. 7SK binds to HMGA1, thus releasing TAR for subsequent interaction with this cofactor [4].

Another mechanism via which HMGA1 influences HIV-1 transcription was uncovered while studying the expression of a reporter gene under the control of viral 5’-LTR from a plasmid [4]. In this case, HMGA1 had a repressive effect. A detailed study of the mechanism of HMGA1 action has shown that it can bind to TAR RNA due to the similarity between its structure and 7SK L2 RNA (*Fig. 9*); HMGA1 may compete with viral protein Tat for binding to TAR RNA. This results in a negative effect of HMGA1 on HIV-1 transcription both in the presence and absence of Tat. The influence of overexpression and knockdown of the *HMGA1* gene and 7SK L2 RNA on transcription from the HIV-1 promoter was studied in the presence and absence of Tat. HMGA1 was found to reduce both the basal and Tat-activated transcription from the HIV-1 promoter, which is partially recovered upon 7SK L2 RNA overexpression. Based on this experiment, the model of HMGA1- mediated repression has been proposed (*Fig. 10*) [4]. According to this model, HMGA1 impedes binding of TAR RNA with Tat, or, in the absence of Tat, with a cellular cofactor of viral transcription that has not been described yet. 7SK L2 RNA competes with TAR for HMGA1, destroys their complex, and takes away the HMGA1 protein from the HIV-1 promoter. This facilitates transcription activation. However, the existence and nature of the putative cellular cofactor involved in this process still remains open.

**Fig. 11:**
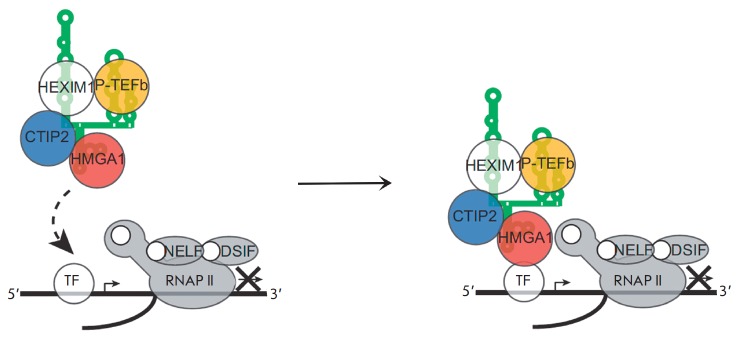
The model of cooperative transcription regulation by HMGA1 and CTIP2. The CTIP2-repressed 7SK/P-TEFb complex is recruited to the promoter through 7SK L2 bound HMGA1 via its interaction with DNA, or with a transcription factor residing in the promoter region [5].

A different model of the repressive effect of HMGA1 on transcription from the HIV-1 promoter has also been proposed [5]. Factors associated with chromatin reorganization play a crucial role in the regulation of HIV-1 transcription; the CTIP2 protein is among these factors. The presence of CTIP2 at the promoter represses transcription of the integrated HIV-1 genome and is typical for the latent state of the virus. CTIP2 recruits histone deacetylases and histone methyltransferases, thus being involved in chromatin condensation [73]. In addition, CTIP2 interacts with the Sp1 and COUP-TF factors by repressing the initial stages of HIV-1 transcription [74] and is involved in delocalization of Tat and its binding to heterochromatin-associated protein HP1 [75]. CTIP2 has recently been shown to interact with 7SK snRNP by binding to loop L2 and the HEXIM1 protein. Within this complex, CTIP2 participates 7SK-L2 HIV-1 TAR *Fig. 9* Structures of the 7SK L2 and TAR RNA regions interacting with HMGA1. The HMGA1 binding site in both RNAs is shown in green. The TAR region responsible for the interaction with Tat and CycT1 is shown in gray [4]. in the repression of Cdk9 kinase that is a component of P-TEFb [76]. It has been discovered that HMGA1 can bind to CTIP2 [5]. Moreover, transcription of a number of cellular genes is negatively regulated by both proteins; some of these genes are transcribed via the P-TEFb/7SK-dependent mechanism [5]. A model of joint transcriptional regulation of these genes by the HMGA1 and CTIP2 proteins has been proposed. It is assumed that HMGA1 can recruit either CTIP2 or the CTIP2/P-TEFb/7SK snRNP complex to the promoters of regulated genes (*Fig. 11*) [5].

It has been demonstrated that when interacting with the HIV-1 promoter, HMGA1 and CTIP2 synergistically repress basal transcription [5]. Knockdown of the* HMGA1 *gene significantly reduces the levels of CTIP2 and P-TEFb/7SK snRNP recruited to the viral promoter and consequently recovers the level of transcription from it. Hence, it is assumed that a mechanism of HMGA1-mediated negative transcriptional regulation similar to that shown in *Fig. 11* may be realized on the viral LTR. Nevertheless, in the case of HIV-1 it remains unclear which DNA region or the LTR-bound factor is involved in recruitment of the HMGA1/CTIP2/7SK snRNP complex. The role of HMGA1 binding to TAR in the HMGA1/CTIP2-mediated repression of basal transcription is also unclear. Neither has it been studied whether HMGA1 has a direct effect on the binding of CTIP2 to viral DNA, similar to what happens with the transcription activator AP-1 [69].

Hence, HMGA1 can both activate and repress HIV- 1 transcription. Its positive effect was seen in induced transcription, while a negative influence was shown for basal transcription. Possibly, some external factors trigger the switch of protein partners of HMGA1 and subsequent alteration in the mechanism of HMGA1- mediated transcriptional regulation.

## CONCLUSIONS

Although the features of HIV-1 transcription have been extensively studied, many aspects still have not been fully elucidated. It is well known that elongation of transcription of the viral genome takes place after the viral regulatory protein Tat binds to TAR RNA, which interacts with the multiprotein transcription elongation factor P-TEFb for its recruitment to the viral promoter. Cyclin-dependent kinase Cdk9 within P-TEFb performs phosphorylation of RNAP II that is required for elongation. However, a question arises: how is the transcription of the latent provirus activated in the absence of Tat protein? It was assumed that the phosphorylation of RNAP II, which is required for eliminating transcription block and transition to the elongation stage, can be activated by cellular factors. Some cellular factors may recruit P-TEFb to the promoter.

Regulation of HIV-1 transcription is a process that involves many cellular proteins; however, the role of some of them is not fully clear. Two cellular proteins described in this review, Ku and HMGA1, are among these “unclear factors.” Data attesting to both the stimulative and repressive effects of these proteins on the expression of HIV-1 genes has been obtained. Their role is often particularly prominent upon basal transcription.

The majority of studies performed on human cells demonstrate that the Ku heterodimer activates transcription from the HIV-1 promoter. The importance of the DNA-PK catalytic subunit for the activation of transcription has been reported in a number of studies. A hypothesis has been put forward that DNA-PK is involved in the transcription elongation stage [10]. Let us mention that the ability of DNA-PK to phosphorylate RNAP II makes this kinase a promising candidate for a protein factor that activates elongation of transcription of viral genes in the absence of Tat.

The architectural factor HMGA1 may influence the chromatin composition upon HIV-1 transcription regulation. In this case, HMGA1 has a positive effect. On the other hand, the interplay between HMGA1 and TAR demonstrated *in vitro *seems to suppress the basal transcription of the HIV-1 genes and is important for maintaining latency [4]. Recruitment of a repressive transcription factor within 7SK snRNP, which HMGA1 can bind to, may be another mechanism of HMGA1-mediated suppression of transcription. There probably is no single mechanism for the involvement of HMGA1 in the regulation of HIV-1 genes transcription; the role of this protein depends on the phase of infection and activity of other cellular proteins. Elucidation of the mechanisms of the influence of Ku and HMGA1 on HIV-1 transcription may result in new approaches for the regulation of the replication of this dangerous virus.

## Abbreviations

HIV-1: human immunodeficiency virus type I
AIDS: acquired immune deficiency syndrome
TAR: trans-activation response element
Tat: trans-activator of transcription
HMGA1: high-mobility group protein A1
DNA-RK: DNA-dependent protein kinase
DNA-PKcs: DNA-PK catalytic subunit
LTR: long terminal repeat
RNAP II: DNA-dependent RNA polymerase II
CTD RNAP II: C-terminal domain of RNAP II
P-TEFb: positive transcription elongation factor b
HTLV-1: human T-lymphotropic virus
MMTV: mouse mammary tumor virus
NRE-1: negative regulatory element 1
TF: transcription factor

## References

[1] van Lint C., Bouchat S., Marcello A. (2013). Retrovirology..

[2] Ruelas D.S., Greene W.C. (2013). Cell..

[3] Zhou M., Halanski M.A., Radonovich M.F., Kashanchi F., Peng J., Price D.H., Brady J.N. (2000). Mol. Cell. Biol..

[4] Eilebrecht S., Wilhelm E., Benecke B.J., Bell B., Benecke A.G. (2013). RNA Biol..

[5] Eilebrecht S., Le Douce V., Riclet R., Targat B., Hallay H., van Driessche B., Schwartz C., Robette G., van Lint C., Rohr O. (2014). Nucleic Acids Research.

[6] Jeanson L., Mouscadet J.F. (2002). J. Biol. Chem..

[7] Masson C., Bury-Moné S., Guiot E., Saez-Cirion A., Schoëvaërt-Brossault D., Brachet-Ducos C., Delelis O., Subra F., Jeanson-Leh L., Mouscadet J.F. (2007). Virology Journal.

[8] Waninger S., Kuhen K., Hu X., Chatterton J.E., Wong-Staal F., Tang H. (2004). Virology Journal.

[9] Manic G., Maurin-Marlin A., Laurent F., Vitale I., Thierry S., Delelis O., Dessen P., Vincendeau M., Leib-Mösch C., Hazan U. (2013). PLoS One..

[10] Tyagi S., Ochem A., Tyagi M. (2011). J. Gen. Virol..

[11] Meyerhans A., Breinig T., Vartanian J.P., Wain-Hobson S. (2003). HIV Sequence Compendium 2003. Los Alamos National Laboratory: Theoretical Biology and Biophysics Group, 2003. 420 p..

[12] Sloan R.D., Wainberg M.A. (2011). Retrovirology..

[13] Colin L., Verdin E., van Lint C. (2014). Meth. Mol. Biol..

[14] Taube R., Peterlin M. (2013). Viruses..

[15] Kwak H., Lis J.T. (2013). Annu. Rev. Genet..

[16] Nguyen V.T., Kiss T., Michels A.A., Bensaude O. (2001). Nature.

[17] Peterlin B.M., Price D.H. (2006). Molecular Cell.

[18] Emiliani S., van Lint C., Fischle W., Paras P. Jr., Ott M., Brady J., Verdin E. (1996). Proc. Natl. Acad. Sci. USA..

[19] Bieniasz P.D., Grdina T.A., Bogerd H.P., Cullen B.R. (1999). Proc. Natl. Acad. Sci. USA..

[20] Yedavalli V.S., Benkirane M., Jeang K.T. (2003). J. Biol. Chem..

[21] Itzen F., Greifenberg A.K., Bösken C.A., Geyer M. (2014). Nucleic Acids Research.

[22] Patel M.C., Debrosse M., Smith M., Dey A., Huynh W., Sarai N., Heightman T.D., Tamura T., Ozato K. (2013). Mol. Cell. Biol..

[23] Jin S., Weaver D.T. (1997). EMBO J..

[24] Ochem A.E., Skopac D., Costa M., Rabilloud T., Vuillard L., Simoncsits A., Giacca M., Falaschi A. (1997). J. Biol. Chem..

[25] Dynan W.S., Yoo S. (1998). Nucleic Acids Research.

[26] Walker J.R., Corpina R.A., Goldberg J. (2001). Nature.

[27] Postow L. (2011). FEBS Lett..

[28] Hill R., Lee P.W. (2010). Cell Cycle..

[29] Fell V.L., Schild-Poulter C. (2015). Mutat. Res. Rev. Mutat. Res..

[30] Yang S.H., Nussenzweig A., Li L., Kim D., Ouyang H., Burgman P., Li G.C. (1996). Mol. Cell. Biol..

[31] Knuth M.W., Gunderson S.I., Thompson N.E., Strasheim L.A., Burgess R.R. (1990). J. Biol. Chem..

[32] Okumura K., Takagi S., Sakaguchi G., Naito K., Minoura-Tada N., Kobayashi H., Mimori T., Hinuma Y., Igarashi H. (1994). FEBS Lett..

[33] Giffin W., Torrance H., Rodda D.J., Préfontaine G.G., Pope L., Hache R.J. (1996). Nature.

[34] Giffin W., Kwast-Welfeld J., Rodda D.J., Préfontaine G.G., Traykova-Andonova M., Zhang Y., Weigel N.L., Lefebvre Y.A., Haché R.J. (1997). J. Biol. Chem..

[35] Giffin W., Gong W., Schild-Poulter C., Haché R.J. (1999). Mol. Cell. Biol..

[36] Shi L., Qiu D., Zhao G., Corthesy B., Lees-Miller S., Reeves W.H., Kao P.N. (2007). Nucleic Acids Research.

[37] Schild-Poulter C., Shih A., Yarymowich N.C., Haché R.J. (2003). Cancer Research.

[38] Jiang D., Zhou Y., Moxley R.A., Jarrett H.W. (2008). Biochemistry..

[39] Wang H., Fang R., Cho J.Y., Libermann T.A., Oettgen P. (2004). J. Biol. Chem..

[40] Sucharov C.C., Helmke S.M., Langer S.J., Perryman M.B., Bristow M., Leinwand L. (2004). Mol. Cell. Biol..

[41] Hill R., Madureira P.A., Waisman D.M., Lee P.W. (2011). Oncotarget..

[42] Mayeur G.L., Kung W.J., Martinez A., Izumiya C., Chen D.J., Kung H.J. (2005). J. Biol. Chem..

[43] Sartorius C.A., Takimoto G.S., Richer J.K., Tung L., Horwitz K.B. (2000). J. Mol. Endocrinol..

[44] Medunjanin S., Weinert S., Schmeisser A., Mayer D., Braun-Dullaeus R.C. (2010). Mol. Biol. Cell..

[45] Lim J.W., Kim H., Kim K.H. (2004). J. Biol. Chem..

[46] Dvir A., Stein L.Y., Calore B.L., Dynan W.S. (1993). J. Biol. Chem..

[47] Maldonado E., Shiekhattar R., Sheldon M., Cho H., Drapkin R., Rickert P., Lees E., Anderson C.W., Linn S., Reinberg D. (1996). Nature.

[48] Mo X., Dynan W.S. (2002). Mol. Cell. Biol..

[49] Peterson S.R., Dvir A., Anderson C.W., Dynan W.S. (1992). Genes Dev..

[50] Ju B.G., Lunyak V.V., Perissi V., Garcia-Bassets I., Rose D.W., Glass C.K., Rosenfeld M.G. (2006). Science..

[51] Studamire B., Goff S.P. (2008). Retrovirology..

[52] Zheng Y., Ao Z., Wang B., Jayappa K.D., Yao X. (2011). J. Biol. Chem..

[53] Santos S., Obukhov Y., Nekhai S., Bukrinsky M., Iordanskiy S. (2012). Retrovirology..

[54] Skalka A.M., Katz R.A. (2005). Cell Death Differ..

[55] Daniel R., Greger J.G., Katz R.A., Taganov K.D., Wu X., Kappes J.C., Skalka A.M. (2004). Virology Journal.

[56] Daniel R., Katz R.A., Merkel G., Hittle J.C., Yen T.J., Skalka A.M. (2001). Mol. Cell. Biol..

[57] Jeanson L., Subra F., Vaganay S., Hervy M., Marangoni E., Bourhis J., Mouscadet J.F. (2002). Virology.

[58] Li L., Olvera J.M., Yoder K.E., Mitchell R.S., Butler S.L., Lieber M., Martin S.L., Bushman F.D. (2001). EMBO J..

[59] Kilzer J.M., Stracker T., Beitzel B., Meek K., Weitzman M., Bushman F.D. (2003). Virology.

[60] Hoover T., Mikovits J., Court D., Liu Y.L., Kung H F., Raziuddin I.O. (1996). Nucleic Acids Research.

[61] Reeves R., Nissen M.S. (1990). J. Biol. Chem..

[62] Ozturk N., Singh I., Mehta A., Braun T., Barreto G. (2014). Front. Cell. Dev. Biol..

[63] Reeves R. (2004). Meth. Enzymol..

[64] Cleynen I., van de Ven W.J. (2008). Int. J. Oncol..

[65] Farnet C.M., Bushman F.D. (1997). Cell..

[66] Hindmarsh P., Ridky T., Reeves R., Andrake M., Skalka A.M., Leis J. (1999). Virology Journal.

[67] Li L., Yoder K., Hansen M.S., Olvera J., Miller M.D., Bushman F.D. (2000). Virology Journal.

[68] Beitzel B., Bushman F. (2003). Nucleic Acids Research.

[69] Henderson A., Bunce M., Siddon N., Reeves R., Tremethick D.J. (2000). Virology Journal.

[70] Henderson A., Holloway A., Reeves R., Tremethick D.J. (2004). Mol. Cell. Biol..

[71] Eilebrecht S., Brysbaert G., Wegert T., Urlaub H., Benecke B.J., Benecke A. (2011). Nucleic Acids Research.

[72] Eilebrecht S., Benecke B.J., Benecke A. (2011). RNA Biol. 2011. V. 8. № 6. 1084–1093..

[73] Marban C., Suzanne S., Dequiedt F., de Walque S., Redel L., van Lint C., Aunis D., Rohr O. (2007). EMBO J..

[74] Marban C., Redel L., Suzanne S., van Lint C., Lecestre D., Chasserot-Golaz S., Leid M., Aunis D., Schaeffer E., Rohr O. (2005). Nucleic Acids Research.

[75] Rohr O., Lecestre D., Chasserot-Golaz S., Marban C., Avram D., Aunis D., Leid M., Schaeffer E. (2003). Virology Journal.

[76] Cherrier T., Le Douce V., Eilebrecht S., Riclet R., Marban C., Dequiedt F., Goumon Y., Paillart J.C., Mericskay M., Parlakian A. (2013). Proc. Natl. Acad. Sci. USA..

